# Toxic Myelitis and Arachnoiditis After Intrathecal Delivery of Bupivacaine via an Implanted Drug Delivery System: Case Report and Review of the Literature

**DOI:** 10.7759/cureus.2240

**Published:** 2018-02-27

**Authors:** Meng Huang, Brian Dalm, Richard K Simpson

**Affiliations:** 1 Department of Neurosurgery, Houston Methodist Neurological Institute

**Keywords:** intrathecal bupivacaine, toxic myelitis, arachnoiditis

## Abstract

The off-label usage of amino-amide anesthetics in intrathecal drug delivery systems (IDDS) for the treatment of chronic non-malignant and malignant pain is supported in the polyanalgesic consensus guidelines as a second-line adjunctive therapy. Although strong evidence for its clinical efficacy is lacking, its clinical safety profile has been well established within established dosing parameters. Despite the rarity of neurological adverse reactions to intrathecal bupivacaine, whether given as regional anesthesia or intrathecal therapy, neurologic morbidity associated with its administration is well documented. The etiology of adverse reactions is often difficult to definitively identify, especially given the variabilities associated with compounding errors in the formulation, solvent contamination, and mechanical factors. We present a rare case of toxic myelitis and arachnoiditis resulting in paraplegia two months after the addition of bupivacaine to the intrathecal analgesic regimen and discuss possible etiological factors with a review of the literature.

## Introduction

The intrathecal drug delivery system (IDDS) is the standard of care for the treatment of chronic pain syndromes and malignant pain refractory to conservative measures. Although the only Food and Drug Administration (FDA) approved intrathecal (IT) analgesic agents at present are morphine and ziconotide, many other medications are used adjunctively in an off-label manner for the treatment of chronic pain [1]. Intrathecal bupivacaine, thought to act at the peripheral nerve, has been popularized for its synergistic properties when combined with centrally acting opioids [2]. Although the efficacy of combined therapy is debatable, it is generally well tolerated [2-5]. Here, we discuss a rare case of presumed toxic myelitis and arachnoiditis after the initiation of intrathecal bupivacaine as an adjunctive agent in a patient with an IDDS.

## Case presentation

A 60-year-old man with chronic lumbar radiculopathy refractory to decompressive laminectomy and failed spinal cord stimulation underwent the placement of a Medtronic Synchromed II 20cc IDDS six months prior to the case presentation. His intrathecal regimen was managed by a community pain specialist and, initially, his pain was very well controlled on intrathecal morphine given at 1.2 mg per day (10 mg/ml). The IDDS data log review revealed that for several months prior to presentation, his pain gradually worsened, prompting a commensurate increase in his intrathecal morphine dosage up to 3.2 mg per day (10 mg/ml). On failure to respond to this dosing, bupivacaine was then supplemented at 3 mg per day (20 mg/ml) two months prior to presentation. Morphine at this time was changed to 25 mg per ml concentration and dosed at 3.8 mg per day. Due to progressive pain and burning dysesthesias in his bilateral lower extremities, his regimen was uptitrated to a dose of 6 mg per day of morphine and 4.8 mg per day of bupivacaine one week prior to presentation. He came to our ED with progressive new lower extremity weakness for 3 days in addition to progressive worsening of burning dysesthesias of both legs. On physical examination, he had 3/5 motor strength throughout the bilateral lower extremities with increased tone, hyperreflexia, 3+ patellar and Achilles tendon reflexes, diminished pain, temperature, light touch, and proprioception below T10 level.

**Figure 1:**
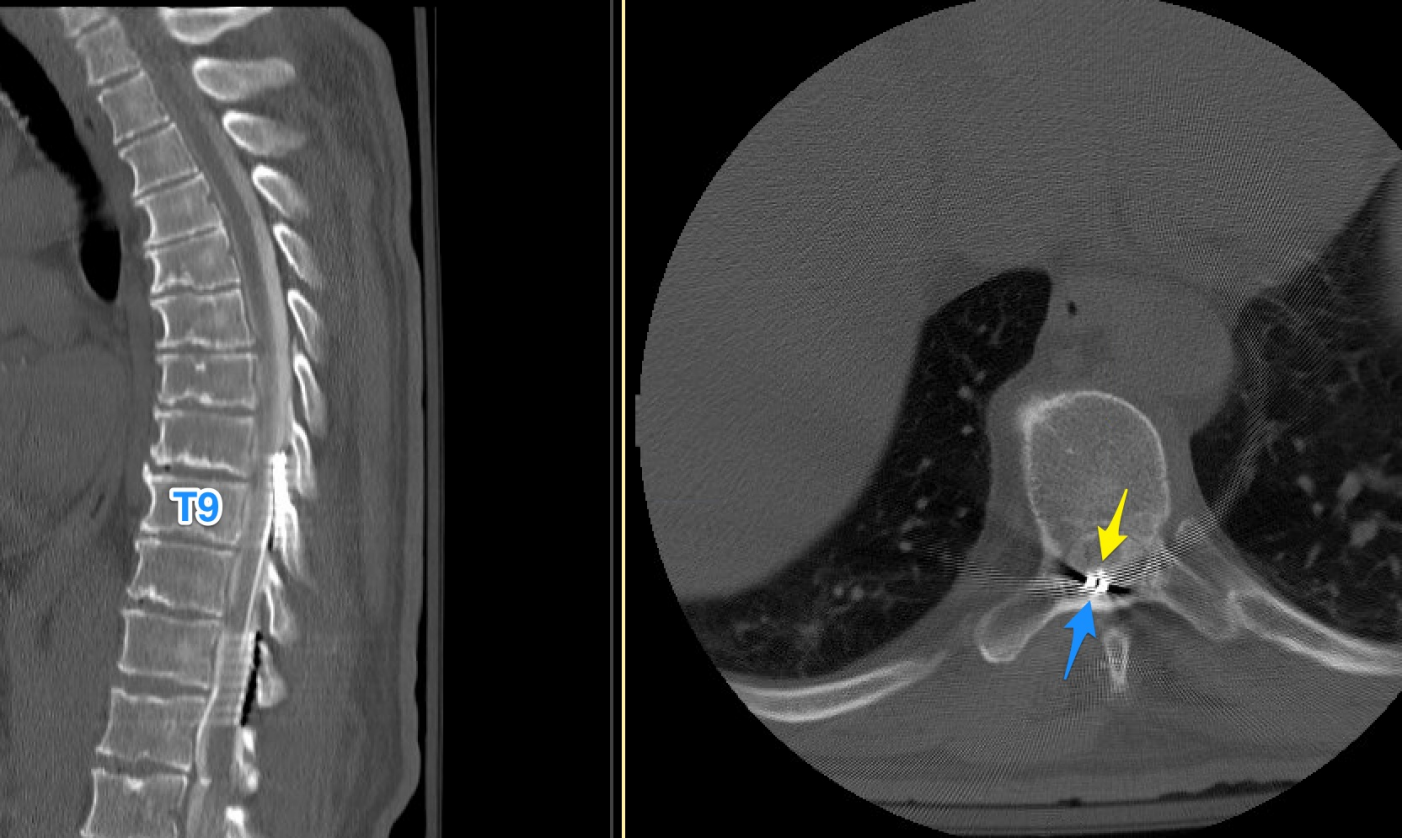
CT myelogram showing the catheter (yellow) and the stimulator lead (blue) tips. No compressive etiology. CT: computed tomography

After admission for a work-up, computed tomography (CT) myelogram showed no compressive etiology (Figure 1).

The tip of the catheter was at the T8-9 level. Cerebrospinal fluid (CSF) studies, despite low glucose levels, showed marked lymphocytic pleocytosis with negative bacterial culture growth, suggestive of inflammatory etiology rather than infections (Table 1).

**Table 1:** CSF analysis showing an inflammatory process with a lymphocytic predominance. CSF: cerebrospinal fluid

CSF Cell Count	748
Red blood cells	306
White blood cells	442
Lymphocytes	90%
Mononuclear cells	3%
Polymorphonuclear	22
Eosinophils	2%
Basophils	2%
Plasma cells	2%
Protein	1154
Glucose	21 (96 blood)
IgG Synthesis Rate	385
Q-Albumin Ratio	220

**Figure 2:**
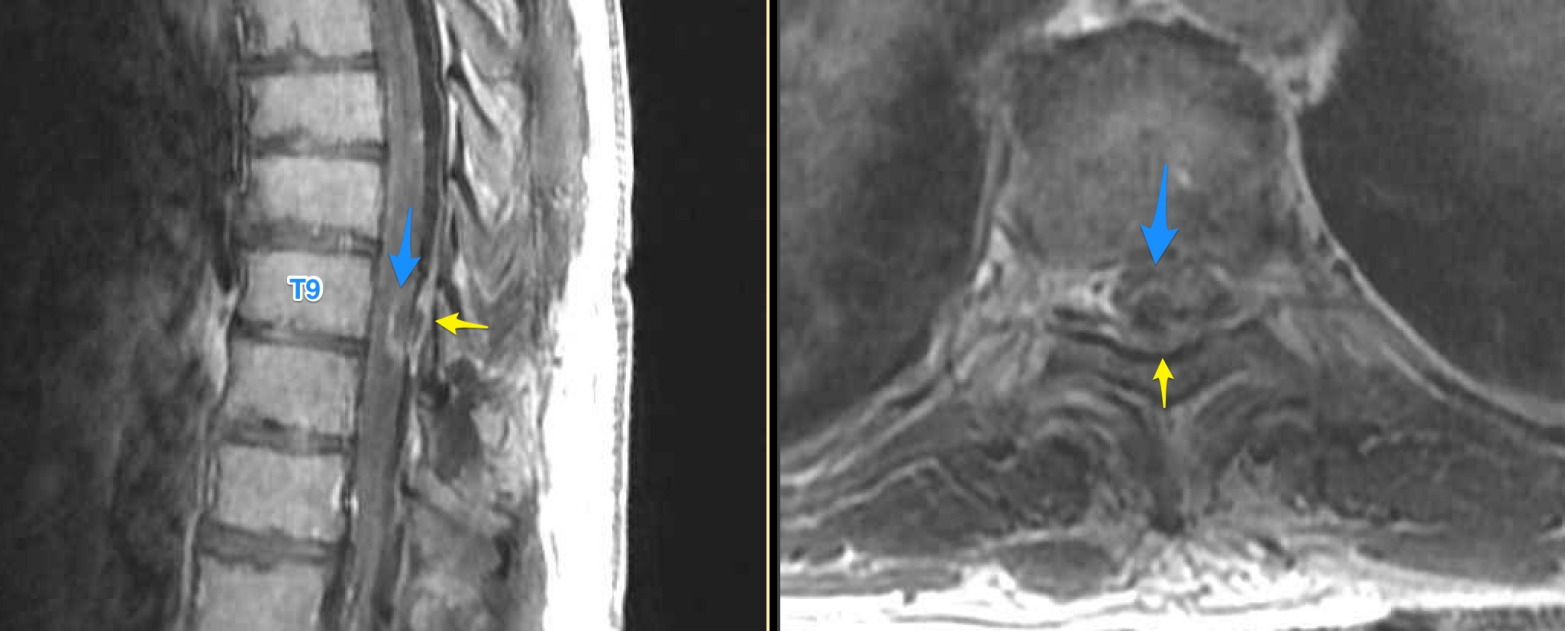
T1 post-contrast sagittal and axial thoracic MRI showing an intramedullary enhancing lesion (blue). Epidural fluid collection (yellow) status post stimulator lead removal is also shown. MRI: magnetic resonance imaging

**Figure 3:**
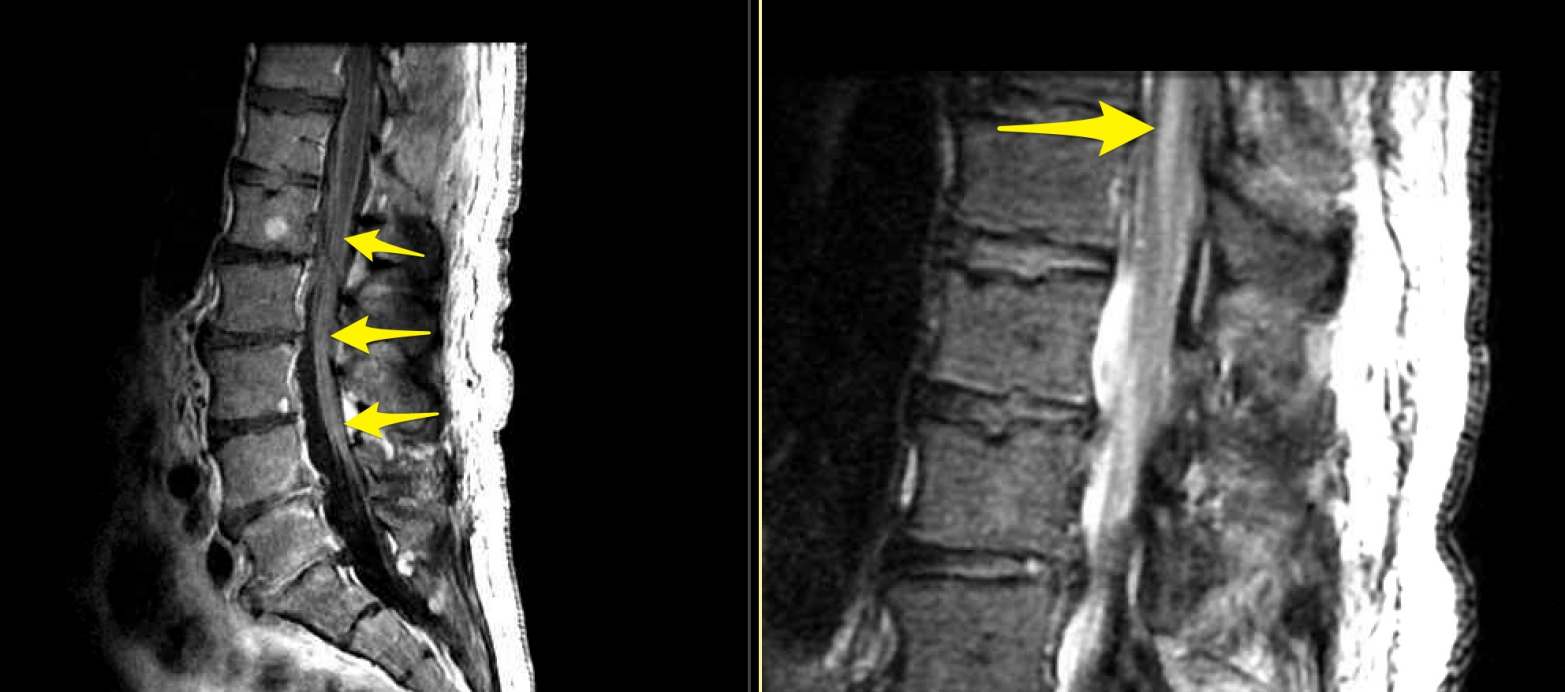
T1 post-contrast sagittal lumbar MRI showing cauda equina enhancement and T2 sagittal lumbar MRI showing conus medullaris edema. MRI: magnetic resonance imaging

The rheumatologic workup revealed no systemic inflammatory processes or markers and the malignancy workup showed no sources of a potential primary on CT chest/abdomen/pelvis. His nontherapeutic spinal cord stimulation (SCS) system was explanted to allow for magnetic resonance imaging (MRI) studies. Pan-spine MRIs showed a noncompressive epidural fluid collection related to precedent stimulator lead removal but did demonstrate T2 signal change from T8 to the conus with a focus of nonexpansive intramedullary enhancement at T8 (Figure 2). There was also diffuse enhancement of the cauda equina (Figure 3).

No tumor-like mass worrisome for drug-induced granuloma was seen [6]. Given the temporal association of his neurologic decline and the increased regimen of intrathecal bupivacaine, bupivacaine neurotoxicity was felt to be the underlying pathophysiologic driver of the myelitis and arachnoiditis. The decision was then made by the patient and all services, including neurology, infectious disease, and pain management, have the contents of the IDDS completely aspirated and replaced with only morphine in an effort to salvage the device. He was concomitantly started on a course of high-dose intravenous steroids. He then began to work with inpatient rehabilitation and was transferred to a rehab facility, where he slowly began to improve and maintained antigravity strength in his lower extremities. A week into inpatient rehabilitation, he experienced acute decline again and was transferred back to our hospital with flaccid paraplegia. His IDDS was explanted for concerns of residual bupivacaine or contaminated carrier, and a new system was implanted and filled with morphine alone. He subsequently convalesced and was transferred back to the rehabilitation center. Long-term follow-up revealed no improvement in his neurologic status.

## Discussion

Regional spinal anesthesia with an intrathecal bolus injection of amino-amide local anesthetics for a dense sensory-motor block has been utilized in clinical practice for over a century, especially in obstetric anesthesia [7-8]. Their reliable clinical efficacy and safety profile has led to the popularization of these agents by pain physicians for use as low-dose, infused, second-line analgesics in implanted drug delivery systems in an FDA off-label manner [1-2]. Amino-amides are hypothesized to act at the dorsal roots, causing the direct inhibition of voltage-gated sodium channels and a conduction block of the peripheral nociceptive A∂ and c fibers. This is in contrast to but adjunctive with the inhibitory effect of opioids on µ2 receptors in the substantia gelatinosa of the spinal cord gray matter [2]. This permits the theoretic advantage of the dosage reduction of individual drug components to minimize adverse effects [3].

The safety and pharmacologic integrity of bupivacaine maintained in an implanted system have been well studied. In 2001, Hildebrand et al. published their evaluation of four Medtronic Synchromed II systems (Medtronic, Minnesota, United States) filled with a commercially available bupivacaine (marcaine) formulation (7.5 mg/ml) of bupivacaine designed for a subarachnoid injection. These systems were maintained at 37 degrees Celsius and samples of fluid were collected from the catheter tip and reservoir at two-week intervals for a total of 12 weeks. The samples were evaluated by liquid chromatography for the concentration of bupivacaine compared to control samples, and all samples showed minimal degradation with greater than 98% concentration. No detectable leaching of titanium was appreciated, and the systems all remained mechanically functional. They also retrospectively reviewed 108 patients who had combination opioid and bupivacaine therapy (2-25 mg/day, average 10 mg/day) with monthly follow-ups for an average of 86 weeks and found no significant adverse events attributable to bupivacaine therapy. Eleven patients developed neurological changes, which after MRI evaluations, were attributed to the progression of the primary disease [4]. A similar study using a stable opioid regimen combined with custom compounded bupivacaine concentrations (0.4-3.7%) for (4-21.4 mg/day) affirms the pharmacologic stability of the agent over time. Of the 12 patients who underwent the prospective trial of efficacy, two patients developed reversible motor weakness at only 5 mg/day total dose whereas the average threshold was 15 mg/day; the overall tolerability was highly variable [8].

Evidence for clinical efficacy is less clear. In a small, retrospective study, intrathecal bupivacaine and diamorphine infused via an external catheter (with a higher flow rate capability than implanted systems) demonstrated significant improvements in malignant extremity pain in six patients followed for 13-87 days [9]. However, in an aforementioned prospective study of 12 patients, no significant improvements in visual analogue scale (VAS) scores were noted during the study period with the addition of bupivacaine up to 21.4 mg/day [8]. Similarly, a double-blinded multicenter crossover study involving 24 patients receiving monthly refills with either 4, 6, or 8 mg/day over 4 months demonstrated no significant changes in the improvement of VAS and disability scores. There was actually a significant placebo effect when patients were enrolled in the study [2]. Although some of these data show that the addition of bupivacaine does not significantly improve the health-related quality of life scores, the stabilization of opioid dosing by the synergistic effect of bupivacaine may be representative of significant overall clinical efficacy. Also, the daily doses of bupivacaine used in the aforementioned studies are relatively small. There are reports of tolerated intrathecal (IT) bupivacaine dosages of up to 90 mg/day [8]. Nonetheless, amino-amides are inherently neurotoxic, and dosing and concentration must be carefully monitored given the lack of strong evidence basis for efficacy and its risk for adverse events. The current consensus guidelines recommend a concentration no greater than 30 mg/ml and no more than 10 mg/day total dosing [1].

Despite the rarity and reversibility of adverse side effects, there are well-known reports of permanent neurologic morbidity associated with intrathecal bupivacaine administration. Most strikingly, with a clinical course that started hyperacutely, a 27-year-old female undergoing regional obstetric anesthesia experienced immediate symptoms of burning pain in her lower extremities within seconds of an injection of 0.5% bupivacaine and 12.5 ug fentanyl. The inflammatory response that ensued over the next days and months resulted in hydrocephalus, requiring ventriculoperitoneal shunting; cervicothoracic syringomyelia, requiring syringo-subarachnoid shunting; and arachnoiditis with cauda equina dysfunction refractory to high-dose intravenous steroids. Ultimately she developed full paraplegia and loss of functional independence. In addition to a gross contamination or misdosing of the drug or its solvent, the authors raised the possibility of chlorhexidine (2% in 70% alcohol) contamination during skin prep as the instigating source of inflammatory stimulus given the immediate symptoms [10].

In our case, the patient did not experience any alarming sensory dysesthesias immediately following the addition of bupivacaine to his IT therapy. Rather, it progressed over several weeks with increasing dosing of morphine and bupivacaine. Sensory symptoms were the first to arise and evolve followed by motor weakness late in the presentation. As such, the clinical course is less suggestive of contamination from skin prep agents. The patient’s IDDS maximum daily dosage of morphine and bupivacaine were within the Pain consensus guidelines maximum of 15mg/day and 10mg/day respectively, making inaccurate dosing less likely assuming no gross error during the drug compounding process. Carrier solvent contamination remains a possibility. Of note, the catheter was immediately juxtaposed to the epidural stimulator electrodes at the rostral end of their ascending course. A possible explanation is that the patient developed epidural fibrosis and adjacent arachnoiditis due to the proximity of these multiple implants, resulting in distal catheter tethering to the dorsal spinal cord and thecal sac. This finding would support a theory that this region of the spinal cord was chronically exposed to a relatively high amount of bupivacaine directly from the catheter tip. In a previous study, the high variability in sensitivity to IT bupivacaine was hypothesized to be associated with catheter position (intraforaminal, cauda equina, spinal cord) [8]. The arachnoiditis may have been a secondary effect of the severe myelitis and central nervous system inflammatory response. Ultimately, the etiology may be a combination of all factors discussed; outpatient clinic records for the patient from the pain management physician and compounding pharmacies were unavailable for review.

## Conclusions

This unfortunate case highlights the potential for severe neurologic morbidity secondary to bupivacaine neurotoxicity. Although the IDDS data logs suggested no deviation from practice guidelines, we hypothesize that multiple spinal surgeries and implants resulted in the local scarring and tethering of the intrathecal catheter to the dorsal thecal sac and spinal cord, resulting in local spinal cord overexposure and toxicity. Pharmaceutical compounding errors and or contamination cannot be ruled out. Although rare, these events can be devastating and, therefore, early cessation of intrathecal bupivacaine and diagnostic workup should be considered in complex cases of refractory pain.
